# A Study of Non-alcoholic Fatty Liver Disease in Patients With Hypothyroidism: A Cross-Sectional Study in a Tertiary Care Hospital

**DOI:** 10.7759/cureus.68956

**Published:** 2024-09-08

**Authors:** Madhulika L Mahashabde, Harin M Bhavsar, Lokesh Kumar, Saketh V Brugumalla

**Affiliations:** 1 General Medicine, Dr. D.Y. Patil Medical College, Hospital, and Research Centre, Pune, IND

**Keywords:** hepatic lipid metabolism, hypothyrodism, hypothyroidism, low-density lipoprotein-cholesterol, non-alcoholic fatty liver diseases (nafld), thyroid-stimulating hormone (tsh)

## Abstract

Background

Hypothyroidism occurs when the thyroid gland is underactive and fails to produce sufficient thyroid hormones. It can affect multiple organs including the heart, brain, liver, kidneys, and reproductive system, leading to symptoms such as fatigue, cognitive impairment, elevated cholesterol, fluid retention, fatty liver, and menstrual irregularities. Given the higher prevalence of fatty liver disease in patients with hypothyroidism, it is important to evaluate the need for routine screening for fatty liver in these patients.

Materials and methods

This observational, cross-sectional study was conducted at Dr. D. Y. Patil Medical Hospital, Pune, Maharashtra, India, from October 2022 to June 2024. The study included 60 patients aged over 12 years who were known or recently diagnosed with hypothyroidism. Patients with type 2 diabetes mellitus, pregnant women, or those with chronic liver disease were excluded. Data collected included physical examination findings and laboratory test results. Fatty liver was diagnosed using magnetic resonance elastography. Statistical analysis was performed using IBM SPSS statistics for Windows, version 20 (IBM Corp., Armonk, New York). The statistical significance of parametric data was evaluated using the Chi-square test. A p-value less than 0.05 and a confidence interval of 95% were considered statistically significant.

Result

The study population had an average age of about 45 years, with most participants aged between 40 and 49 years. The majority of the participants were female, making up over 83% of the group, while males constituted about 17%. The most commonly reported symptom was weight gain, followed by constipation and fatigue. For individuals with fatty liver, the average thyroid-stimulating hormone (TSH) level was notably higher compared to those without fatty liver. Additionally, low-density lipoprotein (LDL) levels were higher in individuals with non-alcoholic fatty liver disease (NAFLD) compared to those without. Both TSH and LDL levels showed a statistically significant association with the occurrence of NAFLD.

Conclusion

Hypothyroidism was more prevalent in females and in the age group 40-49 years. There was a statistical significance between TSH and the occurrence of NAFLD. In this study, statistical significance was also found between LDL and the occurrence of NAFLD.

## Introduction

Hypothyroidism is defined as a clinical condition resulting from the deficiency of thyroid hormones, leading to a generalized slowing down of metabolic processes. Primary hypothyroidism occurs when the thyroid gland cannot produce sufficient thyroid hormones. This can be due to various causes such as autoimmune diseases (like Hashimoto's thyroiditis), iodine deficiency, surgical removal of the thyroid, radiation therapy, or certain medications. Primary hypothyroidism is defined as an increase in thyroid-stimulating hormone (TSH) concentration above the normal level. Primary hypothyroidism can be due to overt or subclinical hypothyroidism. Overt hypothyroidism is characterized by increased TSH levels above the normal range with free thyroxine (T4) levels below the normal range. Increased TSH levels with normal T4 levels characterize subclinical hypothyroidism [1,2]. Secondary hypothyroidism, on the other hand, results from a failure of the pituitary gland to secrete adequate TSH. Causes of secondary hypothyroidism can include pituitary tumors, pituitary surgery or radiation, and other disorders affecting the pituitary gland [3]. In India, the prevalence of hypothyroidism is notably high, affecting approximately 10%-11% of the adult population due to iodine deficiency, autoimmune diseases like Hashimoto's thyroiditis, and genetic predisposition [4]. Hypothyroidism manifests with various symptoms caused by a decrease in metabolic activity. Individuals often experience fatigue, weight gain, sensitivity to cold, constipation, dry skin, hair loss, and facial puffiness [5]. Thyroid hormones primarily affect organs such as the heart, brain, liver, kidneys, and muscles, influencing their metabolism and function.

Thyroid hormones regulate the metabolism of fats, glucose, and proteins, affecting their utilization and storage throughout the body. Thyroid hormones regulate fat metabolism by promoting the breakdown of fats (lipolysis) and protein metabolism by enhancing protein synthesis and degradation, thereby influencing overall energy balance and tissue function [6]. High TSH levels lead to the activation of the PPARα pathway and sterol regulatory element-binding transcription factor 1-c [7]. This promotes hepatic lipogenesis. High TSH levels also decrease hepatic lipoprotein lipase, and this results in the accumulation of triglycerides in the liver [8]. Thyroid hormones influence glucose metabolism by enhancing glucose absorption from the intestines and increasing glucose uptake into cells, thereby helping to regulate blood sugar levels. Thyroid hormones affect the liver by stimulating glycogenolysis (the breakdown of glycogen into glucose) and gluconeogenesis (the production of glucose from non-carbohydrate sources), thereby contributing to the regulation of blood glucose levels [9].

Hypothyroidism is often linked with changes in body weight and body mass index (BMI). When thyroid hormone production is insufficient, it can lead to a decrease in metabolic rate, potentially causing weight gain or difficulty in losing weight. This metabolic slowdown may result from reduced energy expenditure and altered fat and carbohydrate metabolism. Individuals with untreated hypothyroidism may experience an increase in BMI due to these metabolic changes, although the degree of weight gain can vary widely among individuals. Hypothyroidism can impact leptin levels, a hormone involved in regulating appetite and energy expenditure. Studies suggest that hypothyroidism may lead to higher leptin levels due to reduced metabolic activity and altered fat metabolism [10].

Non-alcoholic fatty liver disease (NAFLD) is characterized by steatosis in 5% or more hepatocytes on histopathological examination or 5.6% when diagnosed using magnetic resonance imaging [11]. NAFLD is one of the causes of chronic liver disease. The prevalence of NAFLD in cases of chronic liver diseases is about 25% [12]. Diagnosing NAFLD involves a combination of clinical evaluation and laboratory tests or imaging studies like ultrasound or magnetic resonance elastography. Hypothyroidism is one of the risk factors of NAFLD. The pathogenesis involves reduced thyroid hormone levels leading to decreased lipid metabolism, impaired mitochondrial function, and insulin resistance. These factors contribute to lipid accumulation in the liver, promoting the development of NAFLD [13].

Aim and objectives

The aim and objectives of this study were to know the occurrence of NAFLD in hypothyroid patients, the association of NAFLD with lipid profile and thyroid function test, and to know magnetic resonance elastography findings in hypothyroid patients.

## Materials and methods

Study design and setting

This observational, cross-sectional study was conducted at Dr. D. Y. Patil Medical College, Hospital and Research Centre (DYPMCH), Pune, Maharashtra, India. The study included 60 patients with hypothyroidism between October 2022 and June 2024, with each participant undergoing a thorough clinical assessment and investigation. The study received approval from the Institutional Ethics Committee of DYPMCH (approval number IESC/PGS/2022/14, dated 28/09/ 2022).

Inclusion criteria

Patients with age more than 12 years of age and all patients of hypothyroidism (subclinical and overt) attending IPD and OPD who did not meet the exclusion criteria.

Exclusion criteria

Patients under the age of 12 and pregnant women, patients taking alcohol more than or equal to 10 g/day in women and 20 g/day in men, type 1 diabetes mellitus and type 2 diabetes mellitus, and chronic liver disease.

Sample size

The prevalence of NAFLD in hypothyroidism patients is 27.4% as per the study by Ulla, Ludwig, and Holzner at an acceptable difference of 12%, confidence interval of 95%, and expected loss of subjects of 12%. The sample size is calculated to be 60. The sample size was calculated using WINPEPI version 11.65 (Brixton Health, London, UK) [14].

Data and sample collection

A pro forma was made, which included signs and symptoms of hypothyroidism, other systemic examinations, and physical examination (including calculating the BMI by the metric system (kg/m^2^) and classifying as per Asian Criteria BMI-cutoff). The hemogram was measured using the DxH 900 analyzer (Beckman Coulter, Brea, California), which is based on the Coulter Principle. The Coulter Principle counts the number of cells, cell size, and cell volume by measuring changes in electrical resistance as a particle (such as a cell) passes through a small aperture in a conductive liquid. Renal function tests (RFT) and liver function tests (LFT), along with serum electrolytes, are analyzed using the ARCHITECT c8000 (Abbott, Green Oaks, Illinois) with spectrophotometric measurement methods. The serum used in this study was centrifuged at 4000 rpm for 20 minutes. The serum was excluded in case of being lysed, lipemic, or icteric. Chemiluminescent microparticle immunoassay (CMIA) is a highly sensitive method, which was used in clinical laboratories to assess thyroid function by measuring levels of thyroid hormones and TSH. This involves capturing target hormones with antibodies attached to microparticles, binding with a chemiluminescent molecule, and measuring the emitted light proportional to the concentration. The Abbott Architect ci8200 is an advanced integrated system combining clinical chemistry and immunoassay testing. It can process up to 1200 chemistry and 200 immunoassay tests per hour, using CMIA technology for high sensitivity and specificity.

The sample collection procedure began by identifying and explaining it to the patient. Supplies were gathered, hand hygiene was performed, and gloves were put on. The patient was positioned, a tourniquet was applied, and a venipuncture site was selected. The site was cleaned with an antiseptic and allowed to dry. The needle and holder were assembled, and the needle was inserted into the vein. A blood sample should be collected in an ethylenediaminetetraacetic acid (EDTA) vacutainer (lavender top) for a complete blood count (CBC), LFT, RFT, and Thyroid Function Tests (TFT), and serum electrolytes, following the correct order of drawing to avoid contamination. Magnetic resonance elastography was performed using a 3-tesla magnetic resonance imaging

Data analysis

Data was entered in a Microsoft Excel 2010 (Microsoft Corporation, Redmond, USA) spreadsheet. Data was analyzed in SPSS 29 Grad Pack (IBM SPSS Inc., Chicago, Illinois, USA). Descriptive statistics were calculated including standard deviations, averages, and percentages. The statistical significance of parametric data was evaluated using the Chi-square test. A p-value less than 0.05 and a confidence interval of 95% were considered statistically significant.

## Results

Gender distribution in the study participants

The study group was divided into subjects with fatty liver (Group A) and those without fatty liver (Group B). The gender distribution in both groups was assessed. Among the study group, out of 50 females, 38 (88.3%) had fatty liver, while 12 (11.6%) did not. Out of 10 males, 5 (50%) had fatty liver, while 5 (50%) did not (Table 1).

**Table 1:** Gender distribution in the study participants (N=60) Group A includes participants with fatty liver, and Group B includes participants without fatty liver.

Sex	Group A (N,%)	Group B (N,%)	Total
Female	38 (88.3%)	12 (70.5%)	50
Male	5 (11.6%)	5 (29.5%)	10
Total	43	17	60

Age distribution in the study participants

The age distribution in Group A and Group B was assessed. In the study population, the occurrence of fatty liver was highest in the age group 40-49 years (41.8%), followed by the age groups 30-39 years and 50-59 years, each with 20.9% (Table 2).

**Table 2:** Age distribution across the study participants (N=60) Group A indicates participants with fatty liver, and Group B indicates participants without fatty liver.

Age group (years)	Group A (N,%)	Group B (N,%)	Total
12-20	0 (0%)	0 (0%)	0
20-29	4 (9.3%)	0 (0%)	4
30-39	9 (20.9%)	2 (11.7%)	11
40-49	18 (41.8%)	8 (4.7%)	26
50-59	9 (20.9%)	5 (2.9%)	14
>60	3 (6.9%)	2 (1.1%)	5
Total	43	17	60

Distribution of symptoms in the study participants

The table (Table 3 )compares the distribution of various symptoms between Group A and Group B. The most common symptom in both groups is weight gain, with 18 participants (41.8%) in Group A and 8 participants (47.10%) in Group B experiencing this symptom. The least common symptom in Group A is hair loss, reported by three participants (6.9%), and in Group B, it is hair loss and fatigue, each reported by one participant (5.88%).

**Table 3:** Distribution of symptoms in the study participants in Group A and Group B (N=60) Group A includes participants with fatty liver, and Group B includes participants without fatty liver.

Symptom	Group A (N,%)	Group B (N,%)
Weight gain	18 (41.8%)	8 (47.10%)
Hair loss	3 (6.9%)	1 (5.88%)
Fatigue	5 (11.6%)	1 (5.88%)
Constipation	6 (13.9%)	3 (17.6%)
Asymptomatic	11 (25.5%)	4 (23.5%)

Body mass index distribution in the study participants

In the study group, the occurrence of NAFLD was highest among participants with a BMI of 25-29.9, at 16 (37.2%). Conversely, NAFLD occurrence was lowest in the group with BMI <18.5, at 6.9%. There was a progressive increase in NAFLD occurrence across successive BMI groups, starting from 6.9% in the group with a BMI <18.5 and reaching 37.2% in the group with a BMI of 25-29.9 (Table 4).

**Table 4:** BMI distribution in the study participants (N=60) Group A includes participants with fatty liver, and Group B includes participants without fatty liver. BMI: body mass index

BMI (kg/m^2^)	Group A (N,%)	Group B (N,%)	Total
<18.5	3 (6.9%)	1 (5.88%)	4
18.5-22.9	8 (18.6%)	6 (35.29%)	14
23-24.9	9 (20.9%)	1 (5.88%)	10
25-29.9	16 (37.2%)	6 (35.29%)	22
>30	7 (16.2%)	3 (17.6%)	10
Total	43	17	60

Distribution of hemoglobin in the study participants

The table below (Table 5) presents the distribution of hemoglobin levels among participants in two groups, Group A and Group B, with a total of 60 participants. In Group A, there was one participant (2.3%) with hemoglobin levels below 8 g/dL, whereas Group B had no participants in this category. For hemoglobin levels between 8-10.9 g/dL, Group A had 25 participants (58.13%), and Group B had nine participants (52.9%). In the 11-11.9 g/dL range, Group A had nine participants (20.9%), while Group B had two participants (11.7%). Finally, for hemoglobin levels greater than 12 g/dL, Group A had eight participants (18.6%) compared to Group B, which had six participants (35.29%). Overall, Group A had 43 participants, and Group B had 17 participants, making a total of 60 participants in the study.

**Table 5:** Distribution of hemoglobin among the study participants (N=60) Group A includes participants with fatty liver, and Group B includes participants without fatty liver.

Hemoglobin (g/dL)	Group A (N,%)	Group B (N,%)	Total
<8	1 (2.3%)	0 (0%)	1
8-10.9	25 (58.13%)	9 (52.9%)	34
11-11.9	9 (20.9%)	2 (11.7%)	11
>12	8 (18.6%)	6 (35.29%)	14
Total	43	17	60

Distribution of grades of fatty liver by magnetic resonance elastography in the study participants

In this study, out of 60 study participants, 17 (28%) participants had grade 0 fatty liver, 32 (45%) participants had grade 1 fatty liver, and 11 (18%) participants had grade 2 fatty liver (Table 6).

**Table 6:** Distribution of grades of fatty liver by magnetic resonance plastography in the study participants (N=60) MRI steatosis grading is used to assess the degree of fat accumulation in the liver, typically categorized into different grades based on imaging findings. MRI: magnetic resonance imaging

Magnetic resonance elastography grades of fatty liver	N (%)
0 (No steatosis)	17 (28.3%)
1 (Mild steatosis)	32 (53.3%)
2 (Moderate steatosis)	11 (18.33%)
3 (Severe steatosis)	0 (0%)

Summary of routine laboratory investigations and body mass index among the study participants

In this study, subjects with fatty liver were compared to those without across various laboratory parameters. Subjects with fatty liver exhibited slightly lower hemoglobin levels (10.8±1.53 g/dL) compared to those without fatty liver (11.03±1.9 g/dL). Among other findings, subjects with fatty liver showed higher levels of TSH (38.7±24.3) compared to those without fatty liver (0.5±0.38). Lipid profile parameters such as total cholesterol (180.55±30.52), low-density lipoprotein (LDL) cholesterol (126±18.04), very-low-density lipoprotein (VLDL) cholesterol (21.3±6.83), high-density lipoprotein (HDL) cholesterol (30.8±7.43), and triglycerides (83.04±24.98) were generally higher in subjects with fatty liver compared to those without fatty liver. Additionally, BMI was higher in subjects with fatty liver (25.82±4.79) compared to those without fatty liver (24.97±4.34). These results highlight significant biochemical differences between subjects with and without fatty liver observed in the study (Table 7).

**Table 7:** Summary of routine laboratory investigations and BMI among the study participants (N=60) Group A includes participants with fatty liver, and Group B includes participants without fatty liver. Hb: hemoglobin; TLC: total leukocyte count; AST: aspartate aminotransferase; ALT: alanine aminotransferase; ALP: alkaline phosphatase; TSH: thyroid-stimulating hormone; BMI: body mass index; HDL: high-density lipoprotein; VLDL: very-low-density lipoprotein; LDL: low-density lipoprotein

Laboratory investigation	Reference range	Group A	Group B
Hb	13.2-16.6 g/dL	10.8±1.53 g/dL	11.03±1.9 g/dL
TLC	4000-10000 cells/mm³	7072.1±1651.02 cells/mm³	7276±1676.31 cells/mm³
Platelet	150,000-410,000 /mm³	244,000±49,000 /mm³	227,000±50,000 /mm³
Total bilirubin	0.22-1.2 mg/dL	0.74±0.3 mg/dL	1.04±0.80 mg/dL
Direct bilirubin	<0.5 mg/dL	0.43±0.22 mg/dL	0.56±0.46 mg/dL
Indirect bilirubin	0.1-1.0 mg/dL	0.31±0.15 mg/dL	0.48±0.35 mg/dL
AST	8-48 U/L	35.8±8.2 U/L	38.2±9.29 U/L
ALT	7-55 U/L	45.1±12.2 U/L	42.8±12.15 U/L
ALP	40-129 U/L	88.8±10.3 U/L	91.06±9.14 U/L
TSH	0.35-4.94 µIU/mL	38.7±24.3 µIU/mL	0.5±0.38 µIU/mL
Total cholesterol	<200 mg/dL	180.55±30.52 mg/dL	178.3±22.3 mg/dL
Triglycerides	<150 mg/dL	83.04±24.98 mg/dL	82.24±19.79 mg/dL
HDL	>50 mg/dL (men), >40 mg/dL (women)	30.8±7.43 mg/dL	27.47±5.6 mg/dL
VLDL	<30 mg/dL	21.3±6.83 mg/dL	19.53±7.52 mg/dL
LDL	<100 mg/dL	126±18.04 mg/dL	86.5±22.4 mg/dL
BMI	18.5-22.9 kg/m²	25.82±4.79 kg/m²	24.97±4.34 kg/m²

Association between thyroid-stimulating hormone and the occurrence of non-alcoholic fatty liver disease

In this study, out of 43 patients diagnosed with fatty liver on magnetic resonance elastography, 32 had deranged TSH levels, while 11 had normal TSH levels. Conversely, among the 17 patients without fatty liver on magnetic resonance elastography, three had deranged TSH levels and 14 showed normal TSH levels. The calculated p-value for the association between TSH and the occurrence of NAFLD was 0.01, indicating statistical significance (Table 8).

**Table 8:** Association between TSH and the occurrence of NAFLD (N=60) Group A includes participants with fatty liver, and Group B includes participants without fatty liver. The association between TSH and the occurrence of NAFLD was calculated using the Chi-square test. TSH: thyroid-stimulating hormone; NAFLD: non-alcoholic fatty liver disease

TSH deranged	Group A (N,%)	Group B (N,%)	Total	P-value
Yes	32 (74.4%)	3 (17.6%)	35	0.01
No	11 (25.6%)	14 (82.4%)	25	

Association between low-density lipoprotein and the occurrence of non-alcoholic fatty liver disease

In this study, out of 43 patients diagnosed with fatty liver on magnetic resonance elastography, 24 (55.81%) had deranged LDL levels, and 17 (39.5%) had normal LDL levels. Conversely, among the 17 patients without fatty liver on magnetic resonance elastography, two (11.7%) had deranged LDL levels, while 15 (88.23%) had normal LDL levels. The association between LDL and NAFLD was significant, with a p-value of 0.02 (Table 9).

**Table 9:** Association between LDL and the occurrence of NAFLD (N=60) The association between LDL and the occurrence of NAFLD was calculated using the Chi-square test. LDL: low-density lipoprotein; NAFLD: non-alcoholic fatty liver disease

LDL deranged	Group A (N,%)	Group B (N,%)	Total	P-value
Yes	24 (55.81%)	2 (11.7%)	35	0.02
No	19 (44.1%)	15 (88.23%)	25	

Correlation between thyroid-stimulating hormone and lipid profile

Pearson's correlation coefficient (r) is a statistical measure that quantifies the strength and direction of a linear relationship between two continuous variables. The value of r ranges from -1 to 1, where values closer to 1 indicate a strong positive correlation, values closer to -1 indicate a strong negative correlation, and values near 0 suggest little to no linear relationship. The p-value, on the other hand, measures the statistical significance of the observed correlation. A low p-value (typically <0.05) indicates that the observed correlation is unlikely to have occurred by chance, suggesting that the relationship between the variables is statistically significant. The data reveals a strong positive Pearson's correlation between TSH and LDL levels (r=0.73, p=0.00), indicating that as TSH increases, LDL levels tend to increase significantly. In contrast, there is no significant Pearson's correlation between TSH and Total cholesterol (r=0.06, p=0.64) or TSH and triglycerides (r=0.09, p=0.88), meaning these relationships are weak and not statistically significant. However, a moderate positive Pearson's correlation exists between TSH and VLDL (r=0.34, p=0.01), implying that higher TSH levels are moderately associated with higher VLDL levels, with the p-value indicating this correlation is statistically significant (Table 10).

**Table 10:** Correlation between TSH and lipid profile (N=60) TSH: thyroid-stimulating hormone; LDL: low-density lipoprotein; VLDL: very-low-density lipoprotein

Association	Pearson's correlation coefficient	P-value
TSH and LDL	0.73	0.00
TSH and total cholesterol	0.06	0.64
TSH and triglycerides	0.09	0.88
TSH and VLDL	0.34	0.01

## Discussion

This study aimed to find an association between hypothyroidism and the occurrence of NAFLD. Hypothyroidism is associated with the pathogenesis of NAFLD. Hypothyroidism and NAFLD share overlapping pathogenetic mechanisms. Hypothyroidism can lead to metabolic disturbances, such as dyslipidemia and insulin resistance, which are crucial in the development of NAFLD. Low thyroid hormone levels impair lipid metabolism, causing an increase in serum triglycerides and cholesterol, contributing to hepatic fat accumulation. Thyroid hormones control lipid metabolism by promoting lipolysis and increasing fatty acid oxidation. Thus reduced thyroid hormone production results in the accumulation of lipids inside the liver. Hypothyroidism also results in increased weight and also has a role in metabolic syndrome [15].

Hypothyroidism significantly impacts glucose metabolism by reducing insulin sensitivity and increasing insulin resistance. The thyroid hormones are critical in maintaining normal glucose levels by influencing hepatic glucose production and peripheral glucose uptake. When thyroid hormone levels are low, there is decreased expression of glucose transporters and impaired insulin signaling pathways. This results in reduced glucose uptake by muscles and adipose tissue, leading to higher blood glucose levels. Additionally, hypothyroidism can slow down the rate of glucose absorption in the gastrointestinal tract, further contributing to disrupted glucose homeostasis. Additionally, hypothyroidism also increases insulin resistance, which may lead to NAFLD [9].

Inflammation and oxidative stress are key players in both hypothyroidism and NAFLD pathogenesis. Hypothyroidism induces systemic inflammation and oxidative stress, which can lead to hepatocyte injury and fibrosis in NAFLD. Elevated levels of pro-inflammatory cytokines and reactive oxygen species contribute to liver inflammation and fibrosis, linking hypothyroidism with NAFLD's worsening and progression. Hypothyroidism is also associated with dyslipidemia and increased oxidative stress. As the TSH value increases the risk of NAFLD increases and thus, it is important to diagnose fatty liver in patients with hypothyroidism at an early stage to prevent its progression to advanced liver disease [16].

In this study, hypothyroidism was more prevalent in females. This finding is in accordance with the study done by Vanderpump et al. (2011) [17]. Hypothyroidism was more prevalent in the age group 40-49 years. The most common symptom was weight gain among the study participants. Anemia was present in 44 of the study participants (73.33%). This aligns with the study done by Wopereis et al.(2018) [18]. The association between TSH and NAFLD was statistically significant. Patients with fatty liver had higher values of TSH than patients without fatty liver. This was in accordance with the study conducted by Janovsky et al.(2018)[19]. The association of LDL with the occurrence of NAFLD was also statistically significant per a study done by Sun DQ et al. (2016) [20]. Patients with fatty liver had higher values of LDL than patients without fatty liver. The correlation of TSH with LDL was statistically significant [21].

However, several limitations related to this study must be acknowledged. First, this study being an observational, cross-sectional study assessed the association between hypothyroidism and the occurrence of NAFLD in study participants. However, the occurrence of NAFLD in individuals without hypothyroidism could not be studied. Secondly, there were other factors leading to increased risk of NAFLD for example, dyslipidemia or higher BMI.

## Conclusions

This study provides valuable insights into the complex relationship between hypothyroidism, LDL levels, and the occurrence of NAFLD. Our findings indicate a significant association between hypothyroidism and the presence of NAFLD, suggesting that thyroid dysfunction may contribute to the development of this liver condition. In our study, patients with fatty liver had higher values of TSH than patients without fatty liver. Additionally, we observed that individuals with fatty liver disease exhibit higher LDL levels compared to those without fatty liver, highlighting a potential link between lipid metabolism and NAFLD. These results underscore the necessity of regular thyroid function and lipid profile monitoring in patients at risk for or diagnosed with NAFLD. Understanding these associations can lead to improved diagnostic and therapeutic strategies, ultimately aiding in the effective management and prevention of NAFLD. This study emphasizes the need for further research to explore the underlying mechanisms and potential clinical implications of these findings.
